# MG132 dramatically reduces SAA expression in chicken hepatocellular carcinoma cells at the transcript level independent of its endogenous promoter

**DOI:** 10.1007/s11033-024-09726-9

**Published:** 2024-06-19

**Authors:** Nora-Fabienne Paul, Karolin Gustmann, Jens Tetens, Clemens Falker-Gieske

**Affiliations:** 1 Department of Animal Sciences, Division of Functional Breeding, Georg-August-Universität Göttingen, Burckhardtweg 2, 37077 Göttingen, Germany; 2 Center for Integrated Breeding Research, Georg-August-University, Albrecht-Thaer-Weg 3, 37075 Göttingen, Germany

**Keywords:** Serum amyloid A, SAA, MG132, NF-κB

## Abstract

**Background:**

MG132, a proteasome inhibitor, is widely used to inhibit nuclear factor kappa-light-chain-enhancer of activated B cells (NF-κB) activity by proteasome-mediated degradation of IκB. It has been marketed as a specific, reversible, cell-permeable and low-cost inhibitor. However, adverse effects of the compound have been reported in the literature. We recently discovered and characterised a point mutation in the acute phase protein serum amyloid A (SAA) in chickens, by overexpressing the protein in chicken hepatocellular carcinoma (LMH) cells. This serine to arginine exchange at amino acid position 90 (SAA.R90S) leads to intra- and extracellular accumulation of SAA, which is surprisingly counteracted by MG132 treatment, independent of *SAA*’s intrinsic promoter.

**Methods and Results:**

To test, whether low proteasomal degradation of SAA.R90S is responsible for the observed intra- and extracellular SAA accumulation, we intended to inhibit the proteasome in SAA wild type (SAA.WT) overexpressing cells with MG132. However, we observed an unexpected drastic decrease in SAA protein expression at the transcript level. NF-κB gene expression was unchanged by MG132 at the measured time point.

**Conclusions:**

The observed results demonstrate that MG132 inhibits SAA expression at the transcript level, independent of its endogenous promoter. Further, the data might indicate that NF-κB is not involved in the observed MG132-induced inhibition of SAA expression. We, consequently, question in this brief report whether MG132 should truly be categorised as a specific ubiquitin proteasome inhibitor and recommend the usage of alternative compounds.

**Supplementary Information:**

The online version contains supplementary material available at 10.1007/s11033-024-09726-9.

## Introduction

MG132 (Z-Leu-Leu-Leu-CHO) functions as a potent, rapidly entering, selective and reversible inhibitor of the proteasome and has been used in a variety of research approaches regarding proteasomal degradation [1]. One of its well-known features is the inhibition of nuclear factor kappa-light-chain-enhancer of activated B cells (NF-κB) activation by proteasome-mediated degradation of IκB while maintaining IκB-phosphorylation and ubiquitination [2, 3]. Reports about unwanted behaviour of MG132 in cell culture have been accumulating. These include gene expression changes of essential genes in yeast [4]**,** apoptosis [5–8], cell cycle deregulation [9], and cell toxicity [10].

We recently identified and characterised a dysfunctional mutation in the chicken acute phase protein serum amyloid A (SAA). This mutation is fixed in white egg-laying hens and gives valuable insights into the different susceptibility of amyloid arthropathy in chickens. The onset of amyloid arthropathy is triggered by bacterial infections, and while brown egg-laying chickens are predominantly affected, white egg-laying hens seem to be resistant. Therefore, we established stable cell lines of chicken hepatocellular carcinoma cells overexpressing SAA wildtype (SAA.WT) and SAA carrying the point mutation (SAA.R90S) [11]. *SAA* transcription is induced by LPS and cytokines binding their respective receptors. Signal transduction then initiates the phosphorylation of IκB, allowing to activate the transcription factor NF-κB through an ubiquitin–proteasome pathway. NF-κB, subsequently, is translocated to the nucleus to initiate *SAA* transcription (reviewed here [12]). In our cell culture model, we observed that the SAA.R90S mutation led to intracellular accumulation and extracellular deposition as well as higher aggregation propensity when comparing to SAA.WT [11]. As proteostasis failure is evident in amyloid diseases [13]**,** we wanted to inhibit the ubiquitin–proteasome using MG132. Our cell culture model is not driven by the intrinsic SAA promoter and we therefore expected an increase in the SAA protein level of SAA.WT independent of the endogenous regulation of *SAA* gene expression. To our surprise, MG132 treatment led to a drastic decrease in SAA protein expression at the transcript level.

Here, we provide a brief report on our findings and raise questions about categorising MG132 solely as an ubiquitin proteasome and NF-κB inhibitor.

## Materials and methods

### Cell culture

LMH cell lines overexpressing SAA.WT and SAA.R90S were created in our previous study [11]. Briefly, chicken cDNAs (GgSAA.WT and GgSAA.G270C) were cloned into pCDNA3.1(-) respectively. Stable cell lines were established by lipofection of LMH cells with 2.5 µg of plasmid and subsequent selection with 400 µg/ml G418 (Thermo Scientific). Maintenance was carried out at a concentration of 200 µg/ml G418. 50 µM of proteasome inhibitor MG132 (Fisher Scientific) was supplemented for 24 h. Cells used for western blot analysis were treated as described in our previous study [11]. In brief, cells were detached with accutase and washed with PBS, and pellets were lysed in RIPA buffer. Supernatants of MG132 treated cells were concentrated with Amicon ® Ultra 3K Filter tubes by centrifuging at 4,000 g at 4 °C for 15 min. Samples were normalised to a volume of 200 µL and Halt Protease Inhibitor Cocktail (Fisher Scientific) was added.

### RNA isolation

RNA isolation procedures were described in our previous study [11].

### Real time quantitative PCR

DNA was removed from isolated RNA using DNase I (Thermo Scientific) in accordance with the provided guidelines. Subsequently, qPCR was conducted, with a Rotor-Gene Q (Qiagen), using 50 ng of RNA, following the manufacturer’s protocols for the QuantiNova SYBR Green RT-PCR Kit (Qiagen). The chicken NF-κB forward and reverse primers were 5ʹ-GAAGGAATCGTACCGGGAACA-3ʹ and 5ʹ-CTCAGAGGGCCTTGTGACAGTAA-3ʹ. Primers for chicken SAA and chicken GAPDH were used as in our previous publication [11].

### Western blot

Western blot analysis was performed as described in our previous study, with antibodies for chicken SAA (MCA6030, Bio-Rad, 1:1,000 dilution in EveryBlot) and HRP-conjugated IgG2a (STAR133, Bio-Rad, 1:10,000 dilution in EveryBlot) [11].

### Statistical analyses

Normalised results from qPCR and Western Blot were analysed for statistical significance using RStudio. Comparison between cell lines overexpressing SAA.WT and SAA.R90S was performed using “Analysis of Variance” (ANOVA), followed by the “Compute Tukey Honst Significant Differences” (TukeyHSD) test as post-hoc test.

## Results

**Fig. 1:**
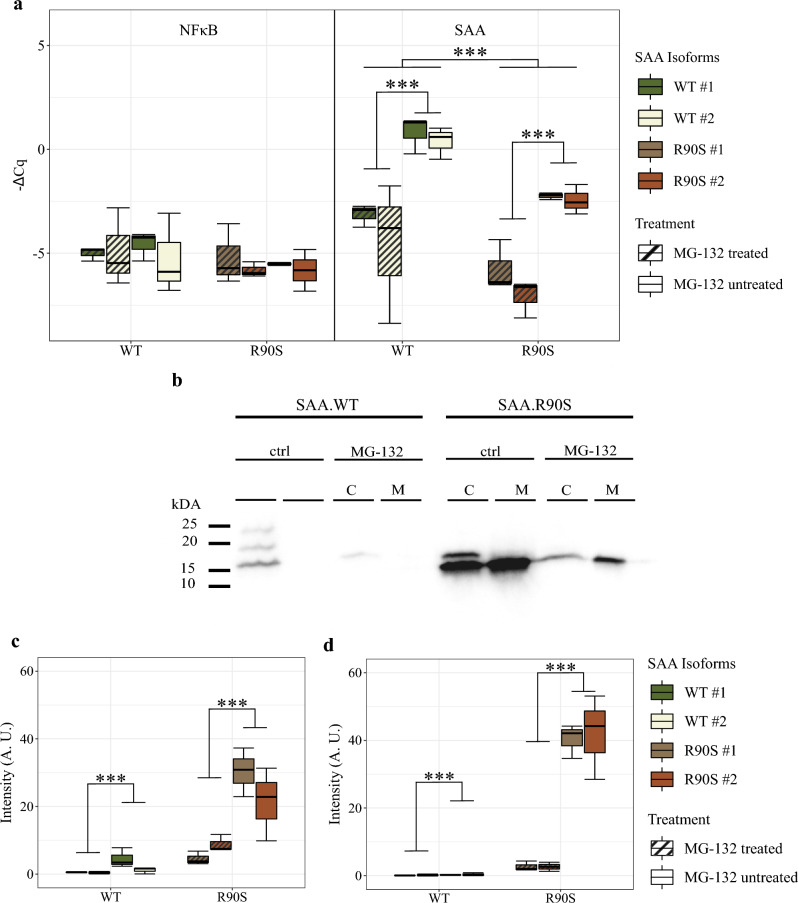
Quantitative transcript analysis of Serum amyloid A (SAA) and nuclear factor kappa-light-chain-enhancer of activated B cells (NF-κB), as well as protein expression analysis of SAA in chicken hepatocellular carcinoma (LMH) cells overexpressing SAA wildtype (SAA.WT) and an SAA isoform harbouring a mutation at position 90, leading to an arginine-serine exchange (SAA.R90S). **a** RT-qPCR analysis of NF-κB and SAA in LMH cells overexpressing SAA.WT and SAA.R90S. **b** and **c** Western blot analysis of SAA protein content in lysates of LMH cells overexpressing SAA.WT and SAA.R90S. **b** and **d** Western blot analysis of SAA protein content in cell culture supernatants of LMH cells overexpressing SAA.WT and SAA.R90S. C = cell lysate; M = Medium

First, we assessed the effects of MG132 at the level of gene transcription by investigating the expression levels of chicken NF-κB and chicken SAA in both MG132-treated and untreated conditions after a 24 h period. The results depicted in Fig. 1a revealed that there were no noteworthy distinctions in the NF-κB expression, neither between the different SAA variants, nor between treated and untreated cells. There were significant differences in the amounts of transcripts between treated and untreated SAA variants (Fig. 1a, *p* adj. = 2.59 × 10^–4^). Interestingly, chicken SAA.WT and SAA.R90S treated with MG132 showed statistically significant decreases in mRNA expression in comparison with respective controls (Fig. 1a, *p* adj. = 2.95 × 10^–4^ and *p* adj. = 8.23 × 10^–4^).

Next, we examined the effects of MG132 at the protein level. When evaluating SAA protein expression of treated and untreated cell lines overexpressing SAA.WT and SAA.R90S, we detected statistically significant decreases in protein levels. SAA levels dropped 6.6-fold within the cells expressing SAA.R90S (Fig. 1b, *p* adj. = 1.4 × 10^–4^) and 15.1-fold in the supernatant of SAA.R90S expressing cells (Fig. 1c, *p* adj. = 2.1 × 10^–6^), after 24h of incubation with MG132. A statistical evaluation between SAA-variants could not be performed, as SAA.WT cells did not express sufficient amounts of SAA protein after MG132 treatment. Raw data and statistical evaluations of the qPCR and Western blot experiments are provided in Supplementary File 1.

## Discussion

The deposition of SAA is a major characteristic of amyloid arthropathy in chickens [14]. We have recently identified a dysfunctional mutation in white egg-laying chickens, which might confer resistance to the disease. Upon characterisation we observed higher levels of SAA.R90S in intra- and extracellular protein content compared to SAA.WT [11]. Defective proteins are degraded by various cellular pathways, e.g. by the proteasome [13, 15]. To prove that SAA.R90S is not degradable by the proteasome, we intended to inhibit the ubiquitin–proteasome with MG132. We expected to see a comparable increase of SAA protein levels in the SAA.WT cells. Protein levels of both SAA isoforms decreased significantly, both intra- and extracellularly (Fig. 1b, c), with MG132 appearing to compensate for the accumulating effect of the SAA.R90S mutation. It has been shown in HC11 mouse mammary epithelial cells that MG132 reverts TNFα induced SAA gene expression on the mRNA level [16]. The authors attributed this effect to the inhibition of NF-κB by MG132, which Nakajima et al. described previously [2]. Indeed NF-κB transcription factor binding sites have been identified in the human *SAA1* and *SAA2* promoter regions [17, 18]. However, since SAA expression in our cell culture model is not driven by the intrinsic SAA promoter, our data questions the theory that SAA downregulation by MG132 is caused by NF-κB inhibition. We here show that MG132 downregulates SAA expression on the transcriptional level. Changes in NF-κB transcript levels were not observable after 24 h of incubation with MG132 (Fig. 1). Although this finding points towards a NF-κB-independent mechanism, additional incubation time points and MG132 concentrations need to be tested to prove this theory. However, incubation times lower than 24 h did not lead to detectable SAA protein quantities in cell culture supernatants. Numerous studies applied a MG132 concentration of 50 µM [19–23], but this is considered the highest recommended concentration by the manufacturer. Hence, cell toxicity and/or apoptosis cannot be ruled out as confounding effects in our observations. Furthermore, monitoring of expression levels and phosphorylation states of p65, p50, and IκBα is warranted to conclusively disprove an involvement of NF-κB in the observed SAA downregulation.

In a transcriptome study in yeast, it was shown that the expression levels of 1028 out of 5716 transcripts were altered after MG132 treatment. Functional clustering of those genes showed an enrichment of genes involved in amino acid biosynthesis, genes involved in cell cycle regulation, and ribosomal protein genes [4]. In light of our results, the observed locus-independent downregulation of *SAA* expression after exposure to MG132 is a remarkable and unexpected occurrence, as we expected an accumulation of SAA.WT protein. We hypothesise that MG132 has an effect on the cell’s transcription machinery or even on the initiation of mRNA degradation, which is consistent with the previously mentioned effects of MG132 in yeast. A second explanation for the observed effect could be inhibition of the CMV promoter that controls SAA expression in our cell culture model. Previous studies have shown the susceptibility of the CMV promoter to suppression by the tumour suppressor protein p53 [24]. In particular, p53 was found to be upregulated in 8B20 murine melanoma cells upon treatment with MG132 [25]. Despite these results, the influence of MG132 on CMV is still unexplored and MG132-mediated disturbance of the cell’s transcription machinery is the more likely scenario in the light of the current state of literature. In order to investigate an effect of MG132 on CMV driven gene expression, previously described effects of MG132 need to be excluded as the cause. These effects include apoptosis [5–8], cell cycle deregulation [9], and cell toxicity [10].

## Conclusions

In summary, MG132 has a broad range of effects on various cellular functions and the results obtained with MG132 should be interpreted with caution. To categorise the compound solely as an ubiquitin proteasome and NF-κB inhibitor is a simplistic and dangerous assumption, as it could influence the study system in unpredictable ways. Especially in light of the fact that MG132 induces apoptosis and is toxic to cells. Therefore, we refrained from further investigation of the effect of MG132 on SAA expression. Future studies should focus on more specific proteasome inhibitors.

## Supplementary Information

Below is the link to the electronic supplementary material.

Supplementary file1 (PDF 310 KB)

## Data Availability

Raw data and programming code are provided in Supplementary File 1.

## Abbreviations

***p adj.***: Adjusted *p* value
**NF-κB**: Nuclear factor kappa-light-chain-enhancer-of-activated B cells
**SAA**: Serum amyloid A
