# Supplementary material for: MG132 dramatically reduces SAA expression in chicken hepatocellular carcinoma cells at the transcript level independent of its endogenous promoter
Source: Mol Biol Rep. 2024 Jun 19;51(1):770. doi: 10.1007/s11033-024-09726-9 (PMC11186868; doi:10.1007/s11033-024-09726-9)
Supplement: Supplementary file 1 — Supplementary file1 (PDF 310 KB) [file 11033_2024_9726_MOESM1_ESM.pdf]

Supporting Information 1: R code for statistical analyses, results of statistical analyses, and normalized results of qPCR and western blot analyses.

Table S1.1: Normalized qPCR results of SAA and NFκB expression in LMH cell lines treated and not treated with MG132.

Cell line	Treated with MG132	-ΔCT SAA	-ΔCT NFκB
SAA.WT.pool 1	x	-3.79	2.81
SAA.WT.pool 1	x	-1.76	5.48
SAA.WT.pool 1	x	-8.37	6.43
SAA.WT.pool 1		-0.47	5.89
SAA.WT.pool 1		1.02	3.07
SAA.WT.pool 1		0.60	6.79
SAA.WT.pool 2	x	-2.92	4.84
SAA.WT.pool 2	x	-2.74	4.79
SAA.WT.pool 2	x	-3.75	5.38
SAA.WT.pool 2		1.37	4.25
SAA.WT.pool 2		1.29	4.10
SAA.WT.pool 2		-0.21	5.37
SAA.R90S.pool 1	x	-6.39	5.72
SAA.R90S.pool 1	x	-6.52	6.34
SAA.R90S.pool 1	x	-4.34	3.58
SAA.R90S.pool 1		-2.10	5.51
SAA.R90S.pool 1		-2.42	5.59
SAA.R90S.pool 1		-2.21	5.44
SAA.R90S.pool 2	x	-8.11	5.95
SAA.R90S.pool 2	x	-6.62	5.41
SAA.R90S.pool 2	x	-6.49	6.09
SAA.R90S.pool 2		-2.55	4.82
SAA.R90S.pool 2		-3.11	5.82
SAA.R90S.pool 2		-1.69	6.83

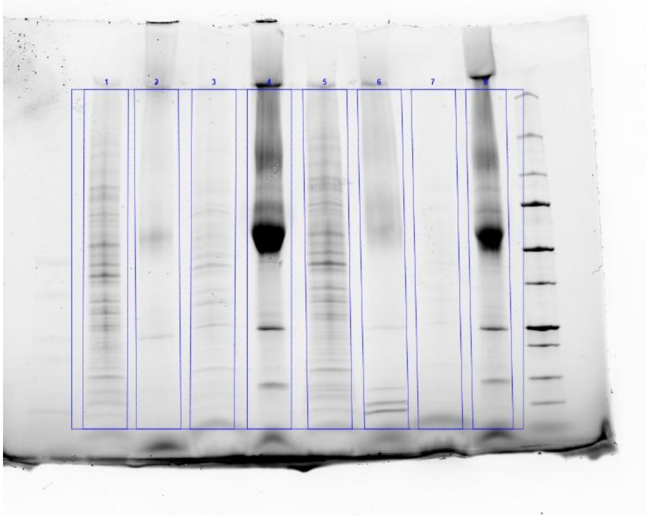

Figure S1.1: 4-15% MP TGX Stain-Free gel of LMH cells overexpressing SAA.WT and SAA.R90S, which were treated and not treated with MG132.

1) LMH SAA.WT cell pellet (untreated)
2) LMH SAA.WT supernatant (untreated)
3) LMH SAA.WT cell pellet (treated with MG132)
4) LMH SAA.WT supernatant (treated with MG132)
5) LMH SAA.R90S cell pellet (untreated)
6) LMH SAA.R90S supernatant (untreated)
7) LMH SAA.R90S cell pellet (treated with MG132)
8) LMH SAA.R90S supernatant (treated with MG132)

Table S1.2: Normalized Western blot results in arbitrary units of SAA protein expression in LMH cell lines, treated with vs without MG132.

Cell line	Treated with MG132	Arbitrary units
SAA.WT.pool 1	x	0.78
SAA.WT.pool 1	x	0.05
SAA.WT.pool 1	x	0.43
SAA.WT.pool 1		1.75
SAA.WT.pool 1		0.06
SAA.WT.pool 1		1.65
SAA.WT.pool 2	x	0.37
SAA.WT.pool 2	x	0.57
SAA.WT.pool 2	x	0.59
SAA.WT.pool 2		3.43
SAA.WT.pool 2		2.33
SAA.WT.pool 2		7.78
SAA.R90S.pool 1	x	6.80
SAA.R90S.pool 1	x	3.08
SAA.R90S.pool 1	x	3.79
SAA.R90S.pool 1		22.91
SAA.R90S.pool 1		30.85
SAA.R90S.pool 1		37.28
SAA.R90S.pool 2	x	11.74
SAA.R90S.pool 2	x	7.25

Table S1.2 (continued): Normalized Western blot results in arbitrary units of SAA protein expression in LMH cell lines, treated with vs without MG132.

Sample Name	Treated with MG132	Arbitrary units
SAA.R90S.pool 2	x	7.47
SAA.R90S.pool 2		9.83
SAA.R90S.pool 2		22.81
SAA.R90S.pool 2		31.33

Table S1.3: Normalized Western blot results in arbitrary units of SAA protein expression in supernatants of LMH cell lines, treated with vs. without MG123.

Cell line	Treated with MG132	Arbitrary units
SAA.WT.pool 1	x	0.49
SAA.WT.pool 1	x	0.01
SAA.WT.pool 1	x	0.05
SAA.WT.pool 1		0.86
SAA.WT.pool 1		0.03
SAA.WT.pool 1		0.14
SAA.WT.pool 2	x	0.04
SAA.WT.pool 2	x	0.05
SAA.WT.pool 2	x	0.09
SAA.WT.pool 2		0.20
SAA.WT.pool 2		0.14
SAA.WT.pool 2		0.31
SAA.R90S.pool 1	x	4.32
SAA.R90S.pool 1	x	1.70
SAA.R90S.pool 1	x	1.98
SAA.R90S.pool 1		42.09
SAA.R90S.pool 1		44.22
SAA.R90S.pool 1		34.67
SAA.R90S.pool 2	x	1.28
SAA.R90S.pool 2	x	2.62
SAA.R90S.pool 2	x	3.96
SAA.R90S.pool 2		53.12
SAA.R90S.pool 2		44.24
SAA.R90S.pool 2		28.45

R code and results of statistical analyses

1) qPCR of SAA expression in LMH cells stably transfected with SAA.WT and SAA.R90S, which were treated and not treated with MG132. Relevant statistical results are in bold.

```
#Anova SAA
library(carData)
library(car)
library(readxl)
SAA <- read_excel("C:/Users/paul43/ownCloud - nora-fabienne.paul@uni-goettingen.de
@owncloud.gwdg.de/Nora/MG-132/7_Statistik_Anova/Anova_Input_Data.xlsx",
                              sheet = "SAA")


SAA$genotype<-as.factor(SAA$genotype)
SAA$bio_rep<-as.factor(SAA$bio_rep)
SAA$treated<-as.factor(SAA$treated)
SAA$exp.rep<-as.factor(SAA$exp.rep)

summary(aov(SAA$signal ~ SAA$genotype + SAA$bio_rep + SAA$treated + SAA$exp.rep))

##               Df Sum Sq Mean Sq F value  Pr(>F)
## SAA$genotype   1  44.89   44.89  21.080 0.00026 ***
## SAA$bio_rep    1   0.06    0.06   0.029 0.86568
## SAA$treated    2 110.08   55.04  25.845   7e-06 ***
## SAA$exp.rep    2   2.11    1.06   0.496 0.61775
## Residuals     17  36.20    2.13
## ---
## Signif. codes:  0 '***' 0.001 '**' 0.01 '*' 0.05 '.' 0.1 ' ' 1

TukeyHSD(aov(SAA$signal ~ SAA$genotype + SAA$bio_rep + SAA$treated + SAA$exp.rep))

##   Tukey multiple comparisons of means
##     95% family-wise confidence level
##
## Fit: aov(formula = SAA$signal ~ SAA$genotype + SAA$bio_rep + SAA$treated + SAA$
exp.rep)
##
## $`SAA$genotype`
##             diff      lwr      upr     p adj
## WT-MUT 2.735375 1.478401 3.992349 0.0002598
##
## $`SAA$bio_rep`
##           diff       lwr      upr     p adj
## 2-1 0.1023126 -1.154662 1.359287 0.8656772
##
## $`SAA$treated`
##                                      diff       lwr       upr     p adj
## MUT_treated-WT_untreated        -4.2782025 -6.673207 -1.883198 0.0004916
## MUT_untreated-WT_untreated      -0.2105947 -2.605599  2.184410 0.9943065
## WT_treated-WT_untreated         -4.4887972 -6.883801 -2.093793 0.0002953
## MUT_untreated-MUT_treated        4.0676079  1.672604  6.462612 0.0008231
## WT_treated-MUT_treated          -0.2105947 -2.605599  2.184410 0.9943065
## WT_treated-MUT_untreated        -4.2782025 -6.673207 -1.883198 0.0004916
##
## $`SAA$exp.rep`
##            diff       lwr      upr     p adj
## 2-1  0.5143554 -1.357513 2.386224 0.7638872
## 3-1 -0.1870730 -2.058941 1.684795 0.9644893
## 3-2 -0.7014284 -2.573297 1.170440 0.6102948
```

2) qPCR of NFκB expression in LMH cells stably transfected with SAA.WT and SAA.R90S treated and not treated with MG132. Relevant statistical results are in bold.

```
#Anova NFKB
library(carData)
library(car)
library(readxl)
NFKB <- read_excel("Anova_Input_Data.xlsx",
                              sheet = "NFKB")
View(NFKB)


NFKB$genotype<-as.factor(NFKB$genotype)
NFKB$bio_rep<-as.factor(NFKB$bio_rep)
NFKB$treated<-as.factor(NFKB$treated)
NFKB$exp.rep<-as.factor(NFKB$exp.rep)

summary(aov(NFKB$signal ~ NFKB$genotype + NFKB$bio_rep + NFKB$treated + NFKB$exp.r
ep))

##                  Df Sum Sq Mean Sq F value Pr(>F)
## NFKB$genotype     1  2.603  2.6032   2.128  0.163
## NFKB$bio_rep      1  0.042  0.0419   0.034  0.855
## NFKB$treated      2  0.075  0.0374   0.031  0.970
## NFKB$exp.rep      2  2.761  1.3804   1.129  0.347
## Residuals        17 20.793  1.2231

TukeyHSD(aov(NFKB$signal ~ NFKB$genotype + NFKB$bio_rep + NFKB$treated + NFKB$exp.
rep))

##   Tukey multiple comparisons of means
##     95% family-wise confidence level
##
## Fit: aov(formula = NFKB$signal ~ NFKB$genotype + NFKB$bio_rep + NFKB$treated +
NFKB$exp.rep)
##
## $`NFKB$genotype`
##              diff       lwr      upr     p adj
## WT-MUT 0.6586871 -0.293905 1.611279 0.1628307
##
## $`NFKB$bio_rep`
##             diff       lwr       upr     p adj
## 2-1 -0.08355474 -1.036147 0.8690374 0.8553732
##
## $`NFKB$treated`
##                                     diff       lwr      upr     p adj
## MUT_treated-WT_untreated         0.05490351 -1.760139 1.869946 0.9997625
## MUT_untreated-WT_untreated      -0.09719229 -1.912235 1.717851 0.9986918
## WT_treated-WT_untreated         -0.04228878 -1.857332 1.772754 0.9998913
## MUT_untreated-MUT_treated       -0.15209579 -1.967139 1.662947 0.9950599
## WT_treated-MUT_treated          -0.09719229 -1.912235 1.717851 0.9986918
## WT_treated-MUT_untreated         0.05490351 -1.760139 1.869946 0.9997625
##
## $`NFKB$exp.rep`
##            diff       lwr       upr     p adj
## 2-1 -0.1015842 -1.520171 1.3170027 0.9815877
## 3-1 -0.7648616 -2.183448 0.6537253 0.3713494
## 3-2 -0.6632774 -2.081864 0.7553095 0.4694769
```

3) Western Blot of SAA expression in LMH cells stably transfected with SAA.WT and SAA.R90S treated and not treated with MG132. Relevant statistical results are in bold.

```
#Anova Test Cell Pellet
library(carData)
library(car)
library(readxl)
Pellet <- read_excel("C:/Users/paul43/ownCloud - nora-fabienne.paul@uni-goettingen
.de@owncloud.gwdg.de/Nora/MG-132/7_Statistik_Anova/Cell pellet/Input Anova Cell ly
sates.xlsx")

Pellet$genotype<-as.factor(Pellet$genotype)
Pellet$bio_rep<-as.factor(Pellet$bio_rep)
Pellet$treated<-as.factor(Pellet$treated)
Pellet$exp.rep<-as.factor(Pellet$exp.rep)

summary(aov(Pellet$signal ~ Pellet$genotype + Pellet$bio_rep + Pellet$treated + Pe
llet$exp.rep))

##                  Df Sum Sq Mean Sq F value   Pr(>F)
## Pellet$genotype   1 1280.8  1280.8  46.105 3.15e-06 ***
## Pellet$bio_rep    1    0.6     0.6   0.023    0.880
## Pellet$treated    2 1116.9   558.5  20.102 3.32e-05 ***
## Pellet$exp.rep    2   71.0    35.5   1.278    0.304
## Residuals        17  472.3    27.8
## ---
## Signif. codes:  0 '***' 0.001 '**' 0.01 '*' 0.05 '.' 0.1 ' ' 1

TukeyHSD(aov(Pellet$signal ~ Pellet$genotype + Pellet$bio_rep + Pellet$treated + P
ellet$exp.rep))

##   Tukey multiple comparisons of means
##     95% family-wise confidence level
##
## Fit: aov(formula = Pellet$signal ~ Pellet$genotype + Pellet$bio_rep + Pellet$tr
eated + Pellet$exp.rep)
##
## $`Pellet$genotype`
##             diff       lwr       upr    p adj
## WT-MUT -14.61063 -19.15046 -10.07079 3.2e-06
##
## $`Pellet$bio_rep`
##           diff       lwr      upr     p adj
## 2-1 -0.3285413 -4.868375 4.211292 0.8804448
##
## $`Pellet$treated`
##                                     diff         lwr       upr     p adj
## MUT_treated-WT_untreated        -10.759410 -19.4094848 -2.109335 0.0122804
## MUT_untreated-WT_untreated        8.389652  -0.2604228 17.039727 0.0590352
## WT_treated-WT_untreated          -2.369758 -11.0198327  6.280317 0.8629912
## MUT_untreated-MUT_treated        19.149062  10.4989871 27.799137 0.0000440
## WT_treated-MUT_treated            8.389652  -0.2604228 17.039727 0.0590352
## WT_treated-MUT_untreated        -10.759410 -19.4094848 -2.109335 0.0122804
##
## $`Pellet$exp.rep`
##         diff       lwr       upr     p adj
## 2-1 1.175671 -5.584986  7.936328 0.8967561
## 3-1 4.091564 -2.669093 10.852221 0.2925045
## 3-2 2.915892 -3.844765  9.676550 0.5231719
```

4) Western Blot of SAA expression in supernatants of LMH cells stably transfected with SAA.WT and SAA.R90S treated and not treated with MG132. Relevant statistical results are in bold.

```
#Anova Test Supernatant
library(carData)
library(car)
library(readxl)
Supernatant <- read_excel("C:/Users/paul43/ownCloud - nora-fabienne.paul@uni-goett
ingen.de@owncloud.gwdg.de/Nora/MG-132/7_Statistik_Anova/Überstand/Input Anova Über
stand.xlsx")

Supernatant$genotype<-as.factor(Supernatant$genotype)
Supernatant$bio_rep<-as.factor(Supernatant$bio_rep)
Supernatant$treated<-as.factor(Supernatant$treated)
Supernatant$exp.rep<-as.factor(Supernatant$exp.rep)

summary(aov(Supernatant$signal ~ Supernatant$genotype + Supernatant$bio_rep + Supe
rnatant$treated + Supernatant$exp.rep))

##                       Df Sum Sq Mean Sq F value   Pr(>F)    
## Supernatant$genotype   1   2822  2821.5 158.369 4.84e-10 ***
## Supernatant$bio_rep    1      1     0.6   0.036    0.851    
## Supernatant$treated    2   4444  2222.0 124.716 6.94e-11 ***
## Supernatant$exp.rep    2     71    35.5   1.993    0.167    
## Residuals             17    303    17.8                     
## ---
## Signif. codes:  0 '***' 0.001 '**' 0.01 '*' 0.05 '.' 0.1 ' ' 1

TukeyHSD(aov(Supernatant$signal ~ Supernatant$genotype + Supernatant$bio_rep + Sup
ernatant$treated + Supernatant$exp.rep))

##   Tukey multiple comparisons of means
##     95% family-wise confidence level
## 
## Fit: aov(formula = Supernatant$signal ~ Supernatant$genotype + Supernatant$bio_
rep + Supernatant$treated + Supernatant$exp.rep)
## 
## $`Supernatant$genotype`
##             diff       lwr       upr p adj
## WT-MUT -21.68536 -25.32096 -18.04976     0
## 
## $`Supernatant$bio_rep`
##          diff       lwr      upr     p adj
## 2-1 0.3285413 -3.307056 3.964139 0.8510506
## 
## $`Supernatant$treated`
##                                    diff        lwr        upr     p adj
## MUT_treated-WT_untreated        -19.3231213 -26.250290 -12.395953 0.0000023
## MUT_untreated-WT_untreated       19.1642282  12.237060  26.091396 0.0000026
## WT_treated-WT_untreated          -0.1588931  -7.086061   6.768275 0.9998963
## MUT_untreated-MUT_treated        38.4873495  31.560181  45.414518 0.0000000
## WT_treated-MUT_treated           19.1642282  12.237060  26.091396 0.0000026
## WT_treated-MUT_untreated        -19.3231213 -26.250290 -12.395953 0.0000023
## 
## $`Supernatant$exp.rep`
##          diff       lwr      upr     p adj
## 2-1 -1.175671 -6.589752 4.238410 0.8442788
## 3-1 -4.091564 -9.505645 1.322518 0.1583365
## 3-2 -2.915892 -8.329974 2.498189 0.3721147
```
